# Lung Cancer With Vertebral Metastases Presenting as Low Back Pain in the Chiropractic Office: A Case Report

**DOI:** 10.7759/cureus.34821

**Published:** 2023-02-09

**Authors:** Eric Chun-Pu Chu, Robert J Trager, Wai Ting Lee, Benjamin Kah Chun Cheong, Steve Yun Ming Hei

**Affiliations:** 1 New York Chiropractic and Physiotherapy Centre, New York Medical Group, Kowloon, HKG; 2 Chiropractic, Connor Whole Health, University Hospitals Cleveland Medical Center, Cleveland, USA

**Keywords:** intervertebral disc displacement, low back pain, adenocarcinoma, neoplasms, lung cancer, chiropractic

## Abstract

Lung cancer commonly metastasizes to the skeletal system, and when affecting the spine, it may initially be mistaken for a typical musculoskeletal source of back pain. We report a previously healthy 52-year-old male non-smoker with an eight-week history of low back pain that radiated into his left thigh and recent weight loss, yet no respiratory symptoms. Initially, the patient visited his primary care physician, who suspected a musculoskeletal condition and prescribed a nonsteroidal anti-inflammatory drug and muscle relaxant, then referred the patient to the chiropractor. Based on the patient’s pain pattern, limited mobility, and other features, the chiropractor suspected a lumbar disc herniation. However, the patient's condition worsened during a one-week trial of care, so the chiropractor ordered magnetic resonance imaging (MRI) and, as the findings suggested vertebral metastasis, promptly referred the patient to an oncologist, who confirmed a diagnosis of lung adenocarcinoma via positron emission tomography (PET)/computed tomography and biopsy. Chiropractors should be aware of warning signs of malignancy, such as unexplained weight loss or progressive worsening despite treatment. If providers suspect spinal metastasis, they should order advanced imaging such as an MRI and refer patients to an oncologist for timely care.

## Introduction

Lung cancer is one of the most prevalent forms of cancer globally [1]. In Hong Kong, the site of the current study, it is the most common type of cancer and the leading cause of cancer-related deaths in men and women [2]. Adenocarcinoma is the most common type of primary lung cancer, accounting for approximately 40% of all cases [3]. Symptoms of lung adenocarcinoma may include cough, shortness of breath, systemic symptoms (e.g., weight loss), or various other symptoms related to potential sites of metastasis (e.g., bone pain) [1].

Lung cancer often has a poor prognosis when it metastasizes [4]. Bone is one of the most common metastatic sites in lung cancer [4,5], with 39% of patients with adenocarcinoma developing bone metastasis [4]. While lung cancer can metastasize throughout the skeletal system, it frequently affects the thoracic and thoracolumbar spine [6]. Pain related to vertebral (spinal) metastasis may begin at a mild intensity and then rapidly worsen [7]. At later stages, vertebral metastasis can manifest with dull, continuous bone pain, pathological fractures, spinal cord compression, cachexia, and hypercalcemia [8].

Chiropractors are healthcare providers who often encounter patients with musculoskeletal complaints, back pain being the most common [9]. Given the potential for spinal metastasis to cause back pain, patients may seek chiropractic providers while being unaware of the serious source of their symptoms. Chiropractors rarely encounter patients with undiagnosed, serious pathology such as cancer. One study in Hong Kong, which included 7,221 adults, found that malignancy was the most common serious undiagnosed pathology that chiropractors encountered, which was found in 0.25% of patients seen for a new episode of low back pain [10]. Despite metastasis being an uncommon presentation in the chiropractic office, it is critical for these providers to promptly recognize and refer such patients to improve their care outcomes.

Here, we describe a rare case of metastasis from lung cancer causing low back pain and presenting to the chiropractic office. Moreover, we emphasize the role of chiropractors in identifying patients with metastases and referring them for appropriate follow-up care.

## Case presentation

Patient information

A 52-year-old man without a previous medical history of illness or significant pain presented to a chiropractor upon referral with an eight-week history of constant low back pain radiating into the left anterior thigh, which he rated 6 out of 10 on the numeric pain rating scale. The patient described his pain as dull and deep and noted that it was exacerbated by transitional movements and lifting. The patient also endorsed having pain at night and noted having severe morning stiffness. The patient was a nonsmoker and social drinker and denied any history of trauma, cough, shortness of breath, or cancer. He acknowledged having an increased nocturnal urinary frequency for over a year, as well as a weight loss of 2 kg over the past month due to a decreased appetite. His father had a myocardial infarction and dementia; however, there was no family history of cancer. The patient worked as an engineer. His World Health Organization Quality of Life score was 86%.

Eight weeks prior, the patient had initially consulted his primary care physician for his back pain, who ordered a metabolic panel and a serum prostate-specific antigen test, both of which were within normal limits. The physician’s initial differential diagnosis included sciatica and lumbar strains. The primary care physician prescribed the patient baclofen, a muscle relaxant, and diclofenac, a nonsteroidal anti-inflammatory medication, which provided some relief. The primary care provider then referred the patient to a chiropractor for conservative management.

Clinical findings

During the initial chiropractic evaluation, the patient was observed to walk with a swayback posture. Palpation revealed restricted intersegmental motion with tenderness at the T7/8, L1/2, L2/3, and L5/S1 spinal segments. Muscular hypertonicity was identified bilaterally in the erector spinae, rectus femoris, and iliopsoas muscles. All active and passive lumbar spine ranges of motion were severely limited and painful. A left lumbar extension-rotation test and straight-leg raise each exacerbated his low back pain with radiation to the left anterior thigh. Motor, sensory, and muscle stretch reflexes were normal.

Given the patient’s symptoms of radiating low back pain and neural tension signs, the chiropractor considered a working diagnosis of lumbar disc herniation with L4 radicular pain. The patient consented to a trial of care with three visits over the span of a week consisting of lumbar flexion-distraction (a gentle type of manipulation used to restore lumbar motion), which only transiently relieved his pain. After one week, a re-examination identified a worsening of the straight leg raise test, with symptoms exacerbated at a lower degree of leg raising. Given the lack of improvement and other red flags (urinary frequency, slight weight loss, pain at night/rest), the chiropractor, therefore, ordered lumbar magnetic resonance imaging (MRI), which the patient obtained that week.

**Figure 1:**
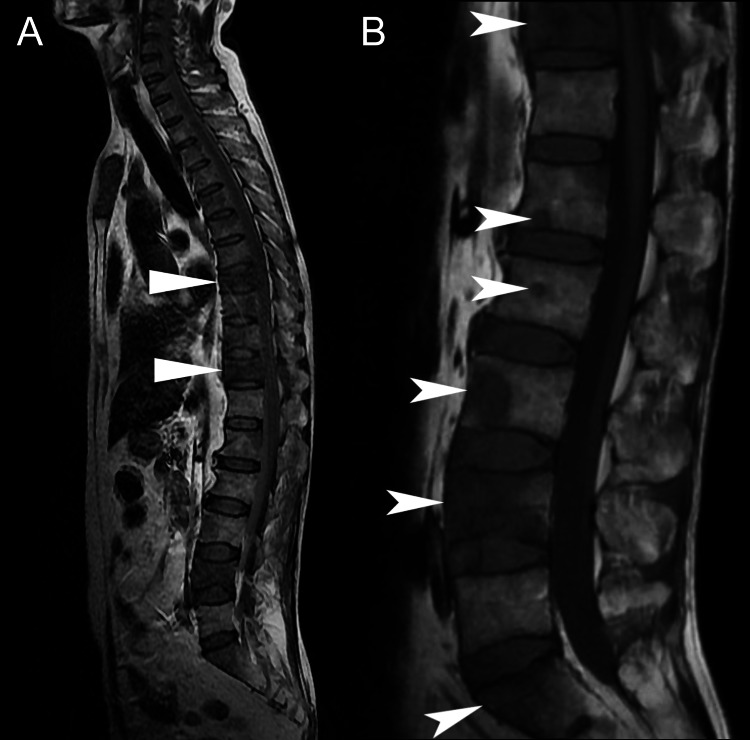
Mid-sagittal magnetic resonance imaging including a scout view (A) and T1-weighted image of the lumbar spine (B) The scout view (A) demonstrates compression fractures of T8 and T11 (triangles), with each level having 20% loss in anterior vertebral body height. In the T1-weighted view (B), heterogeneous marrow signal is evident within multiple vertebral bodies, with hypointense areas indicated in the sacrum, L4, L3, L2, L1, and T11 (arrows), suggestive of spinal metastases. Although there is mild retropulsion at T11 (not indicated), there is no significant narrowing of the central spinal canal.

**Figure 2:**
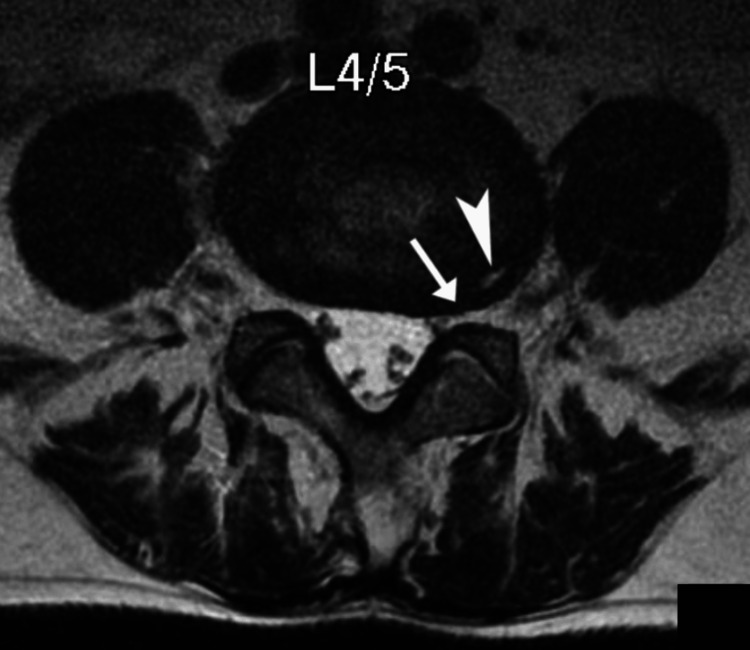
T2-weighted axial lumbar magnetic resonance image at the level of the L4/5 intervertebral disc A left-sided posterior annular fissure (arrowhead) is evident, while a disc bulge impresses the thecal sac and causes mild left neural foraminal stenosis (arrow).

The MRI revealed heterogeneous marrow signals within several vertebral bodies and compression fractures at T8 and T11 (Figure 1). Also noted were degenerative disc changes at L4/5 (Figure 2). As the vertebral fractures and marrow changes were highly suggestive of metastases, the chiropractor promptly referred the patient to an oncologist for further evaluation of suspected metastases.

**Figure 3:**
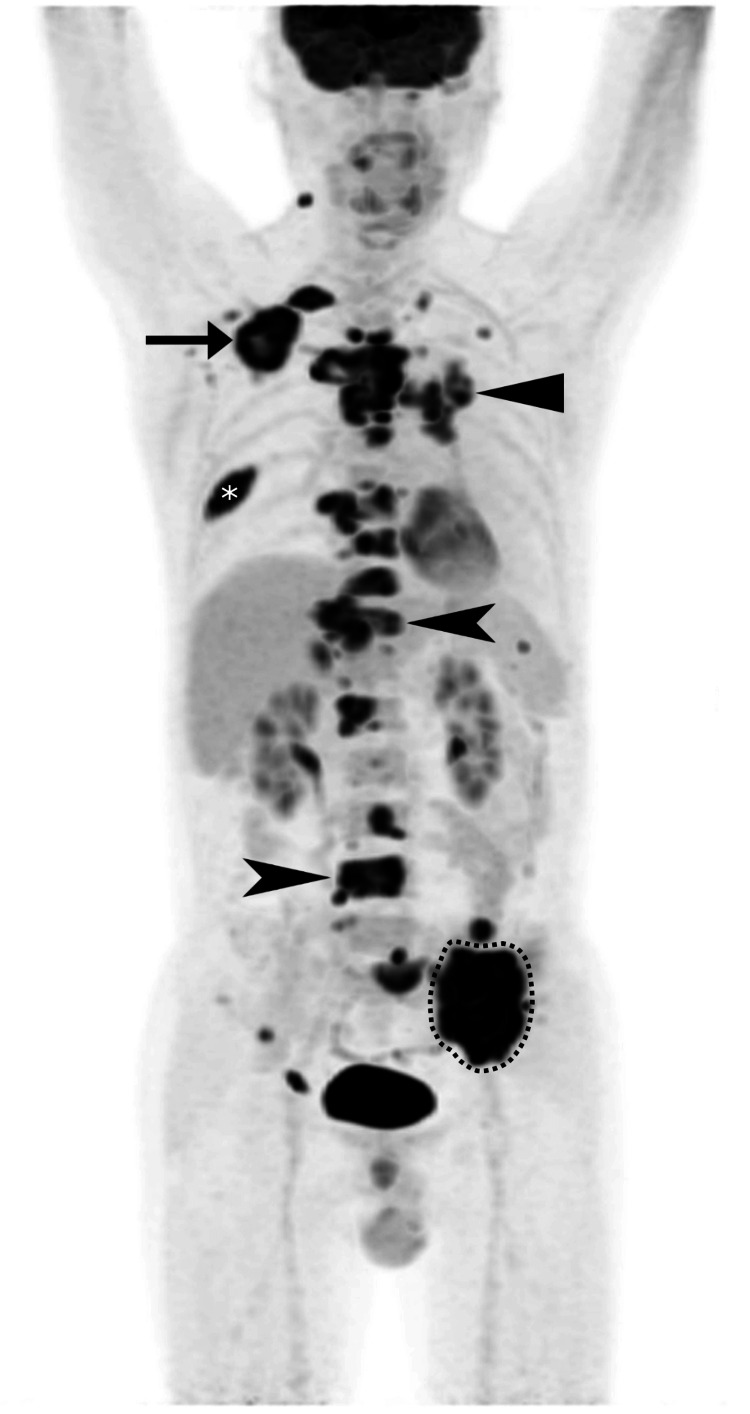
Coronal F18-fluorodeoxyglucose (FDG) whole body positron emission tomography/computed tomography (PET-CT) Increased FDG uptake is evident throughout several sites. Select representative lesions are highlighted with their corresponding maximum standardized uptake values (SUVmax) listed including the vertebrae (arrowheads) T11 (18.01) and L4 (20.42), right scapula (arrow; 20.69), right seventh rib (asterisk; 19.28), left ilium (dotted line; 25.17), and left medial upper lung lobe (triangle; 15.86).

The oncologist conducted an ultrasound-guided biopsy of a right posterior neck triangle lymph node, which was performed using a 16G TemnoTM biopsy needle for four passes. Cytological and histopathological analysis confirmed the diagnosis of adenocarcinoma. F18-fluorodeoxyglucose (FDG) whole-body positron emission tomography/computed tomography (PET-CT) revealed numerous hypermetabolic bone lesions, some of them expansile and lytic, throughout the axial and appendicular skeleton, suggestive of multifocal bony metastases (Figure 3). These involved multiple levels of the cervical to lumbar spine, multiple bilateral ribs, the sternum, the right clavicle, the right scapula, and bilateral pelvic bones. A hypermetabolic subpleural lung mass was also noted at the medial left upper lobe, measuring 2.5 cm × 2.2 cm, consistent with primary lung carcinoma. Hypermetabolic lymph nodes of the left hilum, bilateral mediastinum, and right posterior neck were also evident. The patient was treated via targeted therapy using erlotinib. He provided written consent for the publication of this case report and its related images.

## Discussion

This case describes a 52-year-old man who was referred to a chiropractor for treatment of suspected sciatica by his primary care provider. After a lack of success with a brief trial of care, the chiropractor ordered an MRI, which was suggestive of multiple bone metastases, ultimately leading to a diagnosis of lung adenocarcinoma by an oncologist. The patient’s low back pain can be primarily explained by the presence of vertebral metastases causing cancer-related bone pain. However, his degenerative disc changes at L4/5 may have also contributed to some of his symptoms (i.e., thigh pain) and examination findings (i.e., neural tension), leading to a misleading clinical presentation.

A case-control study found that initial respiratory symptoms were common among those ultimately diagnosed with lung cancer [11]. Cough was the most common presenting symptom, affecting 65% of patients, followed by shortness of breath (56%), and chest or rib pain (42%). In this study, 34% of patients were current or ex-smokers [11]. In contrast, bone pain is less common and occurs in up to 25% of patients, while systemic symptoms such as weight loss are variable in frequency [1]. Our patient did not have any of the common respiratory symptoms of lung cancer and was a non-smoker. Instead, he only had bone pain and systemic symptoms.

This case illustrates the challenges of recognizing metastatic bone disease in the context of what otherwise appears to be a musculoskeletal complaint. As in the current case, radiating low back pain can easily be mistaken for a common degenerative spinal condition [7]. While the current patient did not report any respiratory symptoms related to lung cancer, he did report weight loss, loss of appetite, and increased urinary frequency, which are all red flags for potential serious pathology such as cancer [1,10]. Chiropractors and other healthcare providers should therefore be aware that symptoms of metastasis can be misleading and should conduct further investigation given the presence of red flags or systemic symptoms.

In the current case, the chiropractor’s primary goal was to refer the patient to an oncologist after identifying a potential malignancy via MRI. While a trial of chiropractic treatment is often recommended for low back pain, when the practitioner becomes suspicious of serious pathology, manual treatment should be halted until further investigations are conducted [10]. Fortunately, forceful spinal manipulation was not used in the current case, considering this type of treatment can potentially fracture already-weakened bones [12].

Other cases of patients presenting to a chiropractor with musculoskeletal complaints ultimately caused by lung cancer have been published previously [13-18]. In some of these cases, the patient presented with spinal pain and was eventually diagnosed with skeletal and/or vertebral metastasis [13,14,17,18]. In the current and previous cases, each malignancy was identified using imaging. However, providers should be aware that conventional radiography has a poor sensitivity to detect skeletal metastasis and should consider advanced imaging such as MRI or computed tomography as a first-line investigation when suspecting malignancy [19].

This case has some limitations. We were unable to obtain histology slides or detailed follow-up information, including the response to chemotherapy, as the patient was referred to an outside hospital for treatment. The current case report may not be broadly generalizable. There are regional differences in the scope of chiropractic practice, and it is possible that other chiropractors may not be able to order advanced imaging. Given the patient’s red flag symptoms, ordering an MRI could have been justified even sooner, for example, at the initial chiropractic visit rather than a week later.

## Conclusions

This case describes an adult man who visited a chiropractor with low back pain and was ultimately diagnosed as having skeletal metastasis from adenocarcinoma of the lung. Chiropractors may encounter patients with undiagnosed metastasis who present with back pain. These providers should therefore be vigilant to recognize any warning signs during the initial history-taking and examination and monitor patients’ responses to care. If vertebral metastasis is suspected, providers should request advanced imaging, such as an MRI, and refer the patient to an oncologist for timely care.

## References

[1] Kim J, Lee H, Huang BW (2022). Lung cancer: diagnosis, treatment principles, and screening. Am Fam Physician.

[2] (2023). Overview of cancer statistics in Hong Kong. https://www.cancer.gov.hk/en/hong_kong_cancer/overview_of_cancer_statistics_in_hong_kong.html.

[3] Hutchinson BD, Shroff GS, Truong MT, Ko JP (2019). Spectrum of lung adenocarcinoma. Semin Ultrasound CT MR.

[4] Riihimäki M, Hemminki A, Fallah M, Thomsen H, Sundquist K, Sundquist J, Hemminki K (2014). Metastatic sites and survival in lung cancer. Lung Cancer.

[5] Roato I (2014). Bone metastases: when and how lung cancer interacts with bone. World J Clin Oncol.

[6] Maccauro G, Spinelli MS, Mauro S, Perisano C, Graci C, Rosa MA (2011). Physiopathology of spine metastasis. Int J Surg Oncol.

[7] Börm W, Gleixner M, Klasen J (2004). Spinal tumors in coexisting degenerative spine disease--a differential diagnostic problem. Eur Spine J.

[8] Tsuzuki S, Park SH, Eber MR, Peters CM, Shiozawa Y (2016). Skeletal complications in cancer patients with bone metastases. Int J Urol.

[9] Beliveau PJ, Wong JJ, Sutton DA, Simon NB, Bussières AE, Mior SA, French SD (2017). The chiropractic profession: a scoping review of utilization rates, reasons for seeking care, patient profiles, and care provided. Chiropr Man Therap.

[10] Chu EC, Trager RJ (2022). Prevalence of serious pathology among adults with low back pain presenting for chiropractic care: a retrospective chart review of integrated clinics in Hong Kong. Med Sci Monit.

[11] Hamilton W, Peters TJ, Round A, Sharp D (2005). What are the clinical features of lung cancer before the diagnosis is made? A population based case-control study. Thorax.

[12] Chu EC, Trager RJ, Lee LY, Niazi IK (2023). A retrospective analysis of the incidence of severe adverse events among recipients of chiropractic spinal manipulative therapy. Sci Rep.

[13] Demetrious J, Demetrious GJ (2008). Lung cancer metastasis to the scapula and spine: a case report. Chiropr Osteopat.

[14] Bougie JD, Burns SH (1994). Lung carcinoma presenting as mechanical back pain: a case report. J Can Chiropr Assoc.

[15] Chu EC, Trager RJ, Shum JS, Lai CR (2022). Pancoast tumor presenting as neck pain in the chiropractic office: a case report and literature review. Am J Case Rep.

[16] Downs SE (1990). Bronchogenic carcinoma presenting as neuromusculoskeletal pain. J Manipulative Physiol Ther.

[17] Weiner S, Gardiner L (2001). Pancoast tumor mimicking musculoskeletal pain: a case study. J Neuromusculoskelet Syst.

[18] Cheuvront T, Sergent A (2017). Stage IV small-cell lung cancer presenting as leg pain. Chiropr J Aust.

[19] Shah LM, Salzman KL (2011). Imaging of spinal metastatic disease. Int J Surg Oncol.

